# Mitochondrial DNA in atherosclerosis research progress: a mini review

**DOI:** 10.3389/fimmu.2025.1526390

**Published:** 2025-02-07

**Authors:** Zheng Liu, Nan Huang, Chan Liu, Can Wu, Ling Zhou, Xiang Liu, Haibo Lei

**Affiliations:** ^1^ Department of Clinical Pharmacy, Xiangtan Central Hospital, Xiangtan, China; ^2^ Department of Clinical Pharmacy, Liuyang People’s Hospital, Liuyang, China

**Keywords:** mtDNA, atherosclerosis, mitochondria, inflammation, research progress

## Abstract

Atherosclerosis (AS) is a chronic inflammatory disease that primarily affects large and medium-sized arteries and is one of the leading causes of death worldwide. This article reviews the multifaceted role of mitochondrial DNA (mtDNA) in AS, including its structure, function, release, and relationship with inflammation. Damage and release of mtDNA are considered central drivers in the development of AS, as they participate in the progression of AS by activating inflammatory pathways and affecting lipid metabolism. Therefore, therapeutic strategies targeting mtDNA and its downstream effects may provide new avenues to address this global health challenge.

## 1 Introduction

AS is a chronic inflammatory disease that primarily affects large and medium-sized arteries and is a leading cause of morbidity and mortality worldwide, accounting for approximately 50% of all deaths (1–3). With the changing global lifestyle, the incidence of this disease is gradually increasing, particularly in Western countries. This insidious disease is characterized by the accumulation of lipids, inflammatory cells, and fibrous components within the arterial wall, which, as the disease progresses, can ultimately lead to severe cardiovascular diseases (CVDs), including myocardial infarction, stroke, and peripheral artery disease (4).

AS is driven by lipid accumulation, inflammation, and plaque stability. Endothelial dysfunction initiates the process by allowing LDL-C oxidation and chronic inflammation, which are key in plaque formation (5). This leads to the recruitment of immune cells and the formation of foam cells, which are central to plaque development (6). Oxidized LDL-C further activates immune cells, perpetuating inflammation and plaque progression (7, 8). Plaque stability is determined by factors smooth muscle cell activity, with stable plaques featuring a thick fibrous cap (9). Unstable plaques, with a thinner cap, are prone to rupture, triggering thrombosis and potentially leading to myocardial infarction or stroke (10).

Although scientists have conducted extensive research on the prevention and treatment of AS over the years, the treatment of the disease is still primarily focused on lipid regulation (11). Emerging research indicates that mitochondrial dysfunction, particularly mtDNA, plays a crucial role in the initiation and progression of the disease (12, 13). Studies have identified over 250 diseases closely related to mtDNA, and a wealth of research has demonstrated that mitochondria are involved in various life processes such as human growth, aging, disease, and death (14, 15). The complex interplay between mtDNA mutations, copy number abnormalities, release, and inflammation is increasingly recognized as a central driving factor in AS (13, 16, 17). This article delves into the multifaceted role of mtDNA in AS, summarizing the latest findings on its structure, release, mutations, copy number variations, and their interactions with the development and progression of AS. We further explore the relationship between mtDNA and inflammation, which plays a key role in the formation and progression of atherosclerotic plaques.

## 2 mtDNA

### 2.1 Structure and function of mtDNA

Human mtDNA is a circular double-stranded molecule consisting of 16,569 base pairs, composed of an inner light strand and an outer heavy strand, located within the mitochondrial matrix. It encodes 37 genes that are crucial for mitochondrial function, including 13 mRNAs required for the oxidative phosphorylation (OXPHOS) process, which is the cellular process for energy production (18). Additionally, mtDNA encodes 22 transfer RNAs (tRNAs) and 2 ribosomal RNAs (rRNAs), which are essential for mitochondrial protein synthesis (18, 19). mtDNA is particularly susceptible to damage due to the lack of histone protection and its proximity to the source of reactive oxygen species (ROS) (20). Furthermore, mtDNA lacks effective repair mechanisms, making it more vulnerable to ROS generated during the OXPHOS process (21).

mtDNA possesses three promoter regions, namely the Light Strand Promoter (LSP) region for encoding genes on the L-strand, and the Heavy Strand Promoter 1 (HSP1) and Heavy Strand Promoter 2 (HSP2) regions for encoding genes on the H-strand. These promoter regions are responsible for the simultaneous transcription of multiple genes, producing a single transcript that contains multiple coding sequences (22). Additionally, there is a critical non-coding region within mtDNA, known as the control region or D-loop region, which plays an important role in regulating the transcription and replication processes of mitochondria (23). Due to the higher mutation rate in this region, especially in the hypervariable sequence segments and under conditions of increased oxidative stress, it is particularly susceptible to effects (24). Although these regions do not encode genes, mutations in them can affect the expression levels of the corresponding genes, thereby influencing diseases.

Mitochondria play a crucial role in energy production, calcium homeostasis, and apoptosis regulation. Intact mtDNA is essential for the normal functioning of mitochondria. Therefore, maintaining the integrity of mtDNA is vital for cellular metabolic homeostasis and overall cell survival. Damage to mtDNA can disrupt mitochondrial function, leading to a cascade of detrimental effects (25). If these changes affect genes encoding for OXPHOS, the OXPHOS process will be impaired, and impaired OXPHOS increases ROS production, triggering pro-inflammatory responses and oxidative stress, which are key drivers of AS (26). Moreover, the release of mtDNA into the cytoplasm and extracellular space also has significant impacts on cellular function and the pathogenic microenvironment.

### 2.2 Release of mtDNA

mtDNA is typically confined within the mitochondrial matrix, enclosed in the mitochondrial nucleoid (27), and the presence of mtDNA in the cytoplasm or extracellular space is a result of the loss of mitochondrial integrity. Despite extensive research on this phenomenon in recent years, little is known about the molecular mechanisms that trigger the release of the mitochondrial genome into the extracellular space. Cellular stress can lead to the release of mtDNA into the cytoplasm and extracellular space. It is generally believed that mtDNA release is divided into two modes: active and passive. On one hand, mtDNA can be actively released through specific mechanisms, such as the opening of the mitochondrial permeability transition pore (mPTP) or the formation of mitochondrial-derived vesicles (MDVs); on the other hand, mtDNA can be passively released into the cytoplasm and extracellular space during cell injury, apoptosis, or necrosis (28). Once released, mtDNA acts as a potent damage-associated molecular pattern (DAMP), recognized by pattern recognition receptors (PRRs) such as Toll-like receptor 9 (TLR9) and cyclic GMP-AMP synthase (cGAS). This recognition triggers downstream inflammatory pathways, amplifying the inflammatory environment characteristic of AS (29).

## 3 The role of mtDNA in the regulation of AS

### 3.1 mtDNA mutations and AS

Mitochondrial-related diseases are caused by mutations in mtDNA. However, a mutation in one of the thousands of mitochondria within a cell generally does not lead to disease, as its function can be compensated for by the remaining normal mitochondria. Dynamic mtDNA heteroplasmy determines the clinical severity of mitochondrial diseases. Symptoms only manifest when the threshold of damaged mitochondria reaches 70%-90% (30). An increasing body of evidence suggests that mtDNA mutations are associated with the occurrence and progression of AS (31, 32). These mutations, caused by oxidative stress, environmental damage, or genetic predisposition, typically disrupt genes encoding components of the electron transport chain (ETC), which is the core of OXPHOS (13). This disruption leads to impaired mitochondrial respiration, resulting in a vicious cycle of increased ROS production and further mtDNA damage.

Interestingly, mtDNA mutations are not only associated with mitochondrial diseases. Over the past decades, the impact of population mtDNA mutations has been extensively studied, and it has been linked to the pathophysiological conditions of many diseases, such as aging, cancer, Parkinson’s disease, or CVDs, among others (33–36). Early literature has reported that at least 16 site mutations are closely related to mitochondrial function (36), yet coronary artery disease is often associated with mitochondrial dysfunction (37). Therefore, we speculate that mtDNA mutations may be involved in AS by regulating the oxidative phosphorylation process in mitochondria.

Recent studies suggest that mtDNA mutations may be directly or indirectly linked to AS. Vilne et al. analyzed 265 mt-SNVs in approximately 500,000 British individuals and found certain mtDNA variants were more common in patients with myocardial infarction and/or revascularization (38). A small-sample study from China using high-throughput detection found the A5592G mutation associated with CAD patients and identified two new rare mutations, T5628C and T681C (39). Another Asian study explored the association between mtDNA variants and lipidomic profiles in Chinese coronary heart disease patients, discovering significant correlations between mtDNA variants and traditional blood lipid levels (40). Additionally, a meta-analysis from Japan showed that the m.5178C>A variant in the Japanese population is associated with higher HDL-C and lower LDL-C levels, potentially reducing the risk and extending lifespan for CAD in Japan (41). Despite these studies indicating a causal relationship between mtDNA and AS, including lipid levels as risk factors, large-sample, prospective, multicenter controlled studies are still needed to further elucidate this relationship. Statins, the first-line treatment for AS, may cause muscle symptoms, and research found that Chinese coronary artery disease patients on statins have the m.12630G > A mutation, potentially affecting the prevalence of SAMS (42). These findings suggest that mtDNA mutations may be involved in the development of AS by affecting lipid metabolism.

### 3.2 mtDNA copy number and AS

The number of mtDNA copies in a cell, known as mtDNA-CN, is an indicator of mitochondrial health and biogenesis. A reduction in mtDNA-CN is typically associated with mitochondrial dysfunction and is therefore generally observed to be decreasing in cardiomyocytes from patients with CVDs. Studies have reported that mtDNA-CN levels are negatively correlated with the risk of coronary heart disease (43–45). For instance, researchers have found that mtDNA damage is associated with an increased risk of long-term major adverse cardiac events and all-cause mortality in patients with CAD, emphasizing the importance of mitochondrial dysfunction in AS (46). Concurrently, their findings support the use of mtDNA 4977 deletion and mtDNA-CN as potential prognostic biomarkers for assessing the risk in CAD patients (46). Subsequently, a study by Vasan and colleagues elucidated this relationship. They found that mtDNA-CN has a significant correlation with obesity, hypertension, diabetes, and hyperlipidemia (47).

However, the findings on mtDNA-CN in AS have not yielded a consensus. The results obtained by Liu et al. were in direct contrast to previous studies (48). Yet, they found that low-density lipoprotein cholesterol (LDL-C) has a causal effect on mtDNA-CN. This suggests that the relationship between mtDNA-CN and AS still warrants further exploration, but lipid-lowering may have clinical significance for improving mitochondrial function. The reasons for this phenomenon may be related to differences in the study subjects or the statistical methods used by the researchers.

It is noteworthy that in the ApoE^-/-^ mouse model, a reduction in mtDNA-CN and mitochondrial respiration is associated with an increase in mitochondrial ROS (49). Further validation of the impact of reducing mtDNA damage and increasing mitochondrial respiration on AS was achieved by overexpressing the mitochondrial helicase Twinkle. The results indicated a reduction in the necrotic core area and an increase in the fibrous cap area in atherosclerotic model mice, demonstrating at the animal level that increasing mtDNA-CN may be beneficial for AS (49). A decrease in mtDNA-CN in atherosclerotic plaques is associated with impaired mitochondrial function, increased ROS production, and exacerbated inflammation. The decline in mtDNA-CN may reflect an inability to compensate for mtDNA damage, ultimately impairing cellular energy production and promoting an atherosclerotic environment.

### 3.3 mtDNA damage and mitochondrial dynamics in atherosclerosis

Mitochondrial dynamics, which generally include mitochondrial fusion and fission, are controllers of mitochondrial biogenesis and have been proven to be associated with AS (21, 50). Studies have shown that there is a complex regulatory relationship between mtDNA damage and mitochondrial dynamics. Mitochondrial fusion mainly promotes self-communication and material exchange (mtDNA and proteins), which on one hand can protect intact mtDNA, and on the other hand can compensate for damaged mtDNA, maintaining its normal function (51). Disrupted mitochondrial dynamics can lead to mtDNA damage; moreover, the accumulation of damaged mtDNA can further exacerbate mitochondrial dysfunction, while mitochondrial fission allows the separation of damaged parts of the mitochondria, including damaged mtDNA, through peripheral fission and clearance via the process of mitophagy (52, 53). These processes play an important role in AS.

### 3.4 mtDNA and inflammation in AS

**Figure 1:**
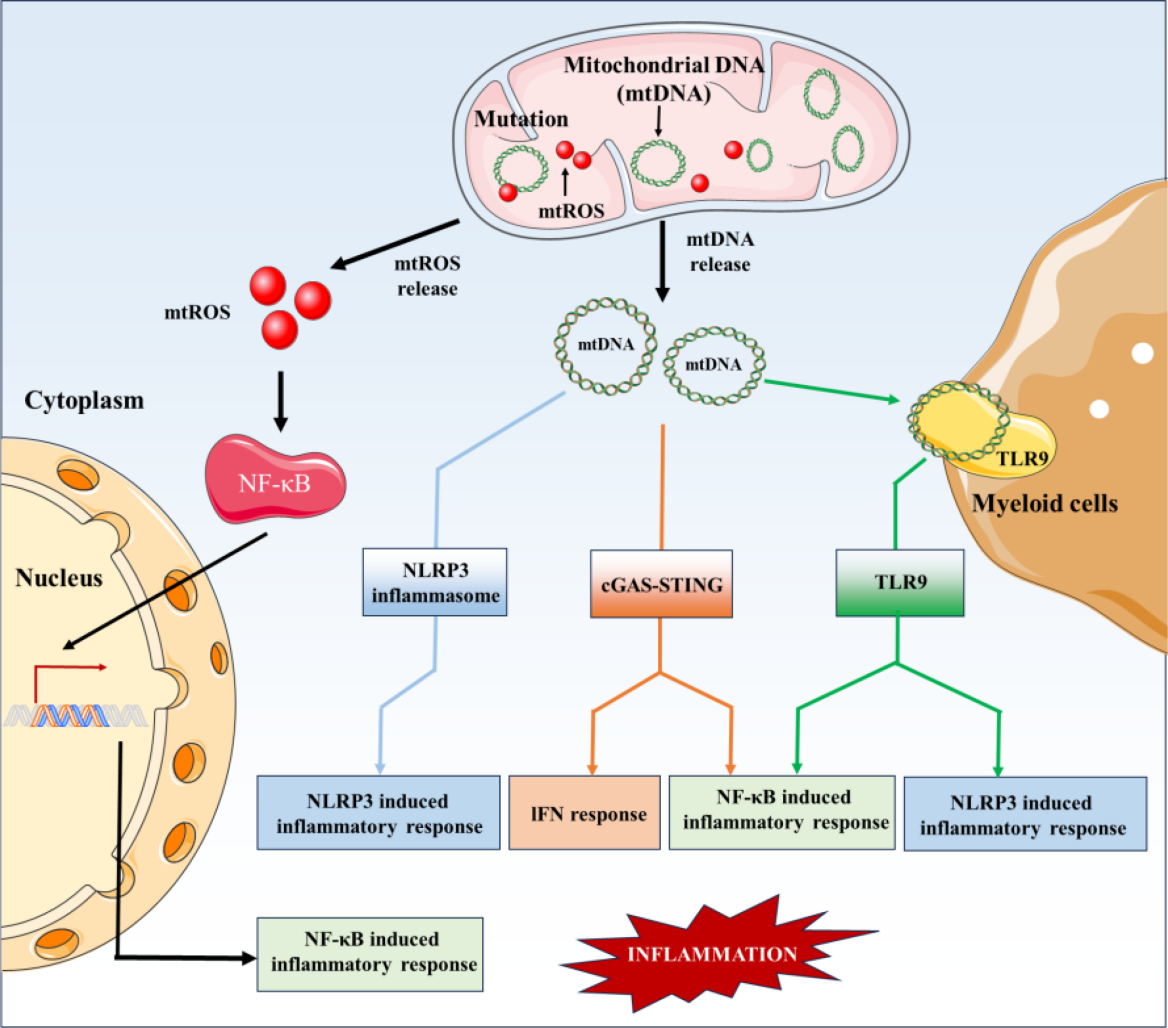
The involvement of mtDNA in regulating inflammatory response in AS.

Inflammation is a hallmark characteristic of AS, and the pro-inflammatory effect of mtDNA was first demonstrated in 2004 (54), suggesting a close relationship between the two. mtDNA itself is a double-stranded circular DNA molecule that, due to its hypo-methylated state and similarity to bacterial DNA, is easily recognized by the immune system as a “foreign” molecule and can trigger various inflammatory pathways. As mentioned earlier, mtDNA released into the cytoplasm or extracellular environment acts as a DAMP, activating PRRs such as TLR9, cGAS, and the NLRP3 inflammasome (28). This activation triggers downstream signaling cascades, leading to the production of pro-inflammatory cytokines and chemokines (such as TNF-α, IL-6, and MCP-1). These signaling molecules perpetuate the inflammatory response, recruit immune cells into the arterial wall, and promote the formation and progression of atherosclerotic plaques. The mechanism by which mtDNA is involved in the regulation of inflammation in AS is shown in **Figure 1**.

TLR9 is an endosomal DNA recognition receptor that can identify both pathogen DNA and self-DNA. Previous studies have demonstrated that mtDNA can activate TLR9-associated inflammatory responses and participate in the regulation of blood lipids (55). Furthermore, in animal and cellular models of AS, damaged mitochondria release mtDNA into the cytoplasm or extracellular environment (56, 57). By recognizing mtDNA in the environment through TLR9 on the cell surface, immune cells in the blood are recruited to the lesion area, thereby triggering the secretion of pro-inflammatory cytokines such as TNF-α and IL-1β, which exacerbate the microenvironment of the lesion area (58). This indicates that the mtDNA-TLR9 axis plays an important role in the development and progression of AS.

The cGAS-STING signaling pathway is responsible for monitoring the abnormal presence of DNA in the cytoplasm and triggering the release of inflammatory factors (59). When cGAS detects abnormal DNA in the cytoplasm, it catalyzes the reaction between guanosine triphosphate and adenosine triphosphate, generating cGAMP molecules, which in turn promote the activation of the immune response. STING, as a cytoplasm-localized protein, can initiate the interferon response by binding to double-stranded DNA or being activated by cyclic dinucleotides. When mtDNA binds to cGAS, cGAS begins to recruit STING, which activates the TANK-binding kinase and NF-κB signaling pathways, leading to the phosphorylation of interferon regulatory factor 3 (IRF3). The activated IRF3 then mediates the transcription of type I and type III interferons and interferon-stimulated genes, initiating an mtDNA-mediated inflammatory response (60). In AS, mtDNA released from damaged endothelial cells leads to the activation of the cGAS-STING pathway, mediating pyroptosis and thus promoting the progression of AS (61, 62). Additionally, the plasma levels of mt-cfDNA may serve as a useful biomarker for AS (61). Therefore, drug development targeting the mtDNA-cGAS-STING pathway shows great potential in the treatment of AS.

A substantial body of literature has demonstrated that the activation of the NLRP3 inflammasome and the inflammatory cascade it induces are detrimental factors in AS (63, 64). In 2011, Nakahira et al. first reported that mtDNA can activate the inflammasome (65). They found that the depletion of autophagy-related proteins leads to mitochondrial dysfunction and accumulation, with these mitochondria producing excessive ROS. Under stimulation with lipopolysaccharide (LPS) or ATP, these mitochondria are more prone to release mtDNA into the cytoplasm, a process that depends on the formation of the NLRP3 inflammasome. Nakahira et al. also proposed that NLRP3 not only acts after the release of mtDNA but may also promote the formation of mPTP on the mitochondria upstream, thereby facilitating the release of mtDNA (65).

Subsequently, researchers reported that in damaged cells, mtDNA is released into the cytoplasm and directly binds to NLRP3 (66, 67). Importantly, NLRP3 appears to have a preference for binding to oxidized mtDNA, which explains the key role of ROS in inflammasome activation (68). Further studies found that the deletion of the autophagy receptor p62 hinders the clearance of damaged mitochondria, exacerbating the formation of the inflammasome and the secretion of IL-1β (69). Recent research has pointed out that newly synthesized and oxidized mtDNA is the main component that binds to NLRP3 (70).

Interestingly, there are differences in the activation of the NLRP3 inflammasome by cytosolic mtDNA and extracellular mtDNA. Unlike cytosolic mtDNA, extracellular mtDNA binds to TLR9 as a DAMP, thereby activating the NLRP3 inflammasome (71). Furthermore, the activation of the NLRP3 inflammasome can further activate the pyroptosis pathway, where the cell membrane is disrupted by GSDMD, increasing the release of inflammatory factors and mtDNA (72). Recently, Miao et al.’s study was the first to report the molecular mechanism by which GSDMD mediates mitochondrial damage (73). Activated GSDMD binds to the phospholipid membrane of mitochondria, forming mitochondrial pores, which disrupts both mitochondrial membranes, leading to the release of mtROS and mtDNA from the mitochondria into the cytoplasm. This evidence all confirms that the positive feedback regulation of mtDNA and NLRP3 is a vicious cycle event in the process of cellular inflammatory necrosis.

In addition to this, mtDNA mutations can also induce and exacerbate inflammation. Impaired OXPHOS caused by mtDNA mutations leads to increased ROS production (74–76). Excessive ROS further activate sensitive transcription factors, such as NF-κB, which is the master regulator of inflammation. The activation of NF-κB amplifies the expression of pro-inflammatory genes, thereby further promoting the chronic inflammatory state characteristic of AS.

## 4 Future directions and conclusion

Although existing studies have shown an association between mtDNA and AS as well as its risk factors, such as blood lipid levels, these studies have limitations, including retrospective design and small sample sizes. Therefore, large-sample, prospective, multicenter controlled studies are needed to more rigorously elucidate the causal relationship between mtDNA and AS.

The multifaceted role of mtDNA in the development of AS suggests that therapeutic strategies targeting mtDNA may have potential for the prevention and treatment of AS. Research on the mechanisms of mtDNA and the development of drugs targeting its inflammatory responses will become a hot topic in future AS prevention and treatment. Given the complex regulatory relationship between mtDNA and oxidative stress and mitochondrial dynamics, targeting oxidative stress and mitochondrial dynamics to regulate mtDNA for the prevention and treatment of atherosclerosis may also become a new perspective for drug development.

In summary, mtDNA has a significant impact on AS due to its structure, function, and characteristics. This article focuses on reviewing the role of mtDNA mutations, copy number, and its regulation of inflammatory responses in the occurrence and development of AS, revealing the potential of mtDNA as a biomarker and therapeutic target in AS.

## References

[1] Malekmohammad K Bezsonov EE Rafieian-Kopaei M. Role of lipid accumulation and inflammation in atherosclerosis: focus on molecular and cellular mechanisms. Front Cardiovasc Med. (2021) 8:707529. doi: 10.3389/fcvm.2021.707529 34552965 PMC8450356

[2] Falk E. Pathogenesis of atherosclerosis. J Am Coll Cardiol. (2006) 47:C7–12. doi: 10.1016/j.jacc.2005.09.068 16631513

[3] Timmis A Aboyans V Vardas P Townsend N Torbica A Kavousi M. European society of cardiology: the 2023 atlas of cardiovascular disease statistics. Eur Heart J. (2024) 45:4019–62. doi: 10.1093/eurheartj/ehae466 39189413

[4] Zhou X Yu L Zhao Y Ge J. Panvascular medicine: an emerging discipline focusing on atherosclerotic diseases. Eur Heart J. (2022) 43:4528–31. doi: 10.1093/eurheartj/ehac448 35947920

[5] Gimbrone MA Jr. García-Cardeña G. Endothelial cell dysfunction and the pathobiology of atherosclerosis. Circ Res. (2016) 118:620–36. doi: 10.1161/CIRCRESAHA.115.306301 PMC4762052 26892962

[6] Gui Y Zheng H Cao RY. Foam cells in atherosclerosis: novel insights into its origins, consequences, and molecular mechanisms. Front Cardiovasc Med. (2022) 9:845942. doi: 10.3389/fcvm.2022.845942 35498045 PMC9043520

[7] Tsimikas S Miller YI. Oxidative modification of lipoproteins: mechanisms, role in inflammation and potential clinical applications in cardiovascular disease. Curr Pharm Design. (2011) 17:27–37. doi: 10.2174/138161211795049831 21226665

[8] Gisterå A Ketelhuth DFJ. Lipid-driven immunometabolic responses in atherosclerosis. Curr Opin Lipidology. (2018) 29:375–80. doi: 10.1097/MOL.0000000000000540 30156570

[9] Chistiakov DA Orekhov AN Bobryshev YV. Contribution of neovascularization and intraplaque haemorrhage to atherosclerotic plaque progression and instability. Acta physiologica (Oxford England). (2015) 213:539–53. doi: 10.1111/apha.2015.213.issue-3 25515699

[10] Kawai K Kawakami R Finn AV Virmani R. Differences in stable and unstable atherosclerotic plaque. Arteriosclerosis thrombosis Vasc Biol. (2024) 44:1474–84. doi: 10.1161/ATVBAHA.124.319396 38924440

[11] Quispe R Sweeney T Varma B Agarwala A Michos ED. Recent updates in hypertriglyceridemia management for cardiovascular disease prevention. Curr Atheroscl Rep. (2022) 24:767–78. doi: 10.1007/s11883-022-01052-4 35895246

[12] Ji X Guo W Gu X Guo S Zhou K Su L. Mutational profiling of mtDNA control region reveals tumor-specific evolutionary selection involved in mitochondrial dysfunction. EBioMedicine. (2022) 80:104058. doi: 10.1016/j.ebiom.2022.104058 35594659 PMC9121266

[13] Khotina VA Vinokurov AY Sinyov VV Zhuravlev AD Popov DY Sukhorukov VN. Mitochondrial dysfunction associated with mtDNA mutation: mitochondrial genome editing in atherosclerosis research. Curr Medicinal Chem. (2024). doi: 10.2174/0109298673323639240926095549 39400015

[14] Zhang L Wu J Zhu Z He Y Fang R. Mitochondrion: a bridge linking aging and degenerative diseases. Life Sci. (2023) 322:121666. doi: 10.1016/j.lfs.2023.121666 37030614

[15] Harrington JS Ryter SW Plataki M Price DR Choi AMK. Mitochondria in health, disease, and aging. Physiol Rev. (2023) 103:2349–422. doi: 10.1152/physrev.00058.2021 PMC10393386 37021870

[16] Yu EP Bennett MR. Mitochondrial DNA damage and atherosclerosis. Trends Endocrinol metabolism: TEM. (2014) 25:481–7. doi: 10.1016/j.tem.2014.06.008 25034130

[17] Natarajan N Florentin J Johny E Xiao H O’Neil SP Lei L. Aberrant mitochondrial DNA synthesis in macrophages exacerbates inflammation and atherosclerosis. Nat Commun. (2024) 15:7337. doi: 10.1038/s41467-024-51780-1 39187565 PMC11347661

[18] Wallace DC. Mitochondrial genetic medicine. Nat Genet. (2018) 50:1642–9. doi: 10.1038/s41588-018-0264-z 30374071

[19] Chocron ES Munkácsy E Pickering AM. Cause or casualty: The role of mitochondrial DNA in aging and age-associated disease. Biochimica et biophysica acta. Mol Basis Dis. (2019) 1865:285–97. doi: 10.1016/j.bbadis.2018.09.035 PMC6310633 30419337

[20] Wang L Zhang Q Yuan K Yuan J. mtDNA in the Pathogenesis of Cardiovascular Diseases. Dis Markers. (2021) 2021:7157109. doi: 10.1155/2021/7157109 34795807 PMC8595034

[21] Markin AM Khotina VA Zabudskaya XG Bogatyreva AI Starodubova AV Ivanova E. Disturbance of mitochondrial dynamics and mitochondrial therapies in atherosclerosis. Life (Basel Switzerland). (2021) 11:165. doi: 10.3390/life11020165 33672784 PMC7924632

[22] Mposhi A van der Wijst MG Faber KN Rots MG. Regulation of mitochondrial gene expression, the epigenetic enigma. Front Bioscience (Landmark edition). (2017) 22:1099–113. doi: 10.2741/4535 28199194

[23] Zhang R Zhang F Wang C Wang S Shiao YH Guo Z. Identification of sequence polymorphism in the D-Loop region of mitochondrial DNA as a risk factor for hepatocellular carcinoma with distinct etiology. J Exp Clin Cancer Research: CR. (2010) 29:130. doi: 10.1186/1756-9966-29-130 20849651 PMC2949825

[24] Todosenko N Khaziakhmatova O Malashchenko V Yurova K Bograya M Beletskaya M. Mitochondrial dysfunction associated with mtDNA in metabolic syndrome and obesity. Int J Mol Sci. (2023) 24:1–20. doi: 10.3390/ijms241512012 PMC10418437 37569389

[25] Ding Z Liu S Wang X Dai Y Khaidakov M Romeo F. LOX-1, oxidant stress, mtDNA damage, autophagy, and immune response in atherosclerosis. Can J Physiol Pharmacol. (2014) 92:524–30. doi: 10.1139/cjpp-2013-0420 24959993

[26] Hulsmans M Holvoet P. The vicious circle between oxidative stress and inflammation in atherosclerosis. J Cell Mol Med. (2010) 14:70–8. doi: 10.1111/j.1582-4934.2009.00978.x PMC3837590 19968738

[27] Farge G Falkenberg M. Organization of DNA in Mammalian Mitochondria. Int J Mol Sci. (2019) 20:1–14. doi: 10.3390/ijms20112770 PMC6600607 31195723

[28] De Gaetano A Solodka K Zanini G Selleri V Mattioli AV Nasi M. Molecular Mechanisms of mtDNA-Mediated Inflammation. Cells. (2021) 10:1–21. doi: 10.3390/cells10112898 PMC8616383 34831121

[29] Riley JS Tait SW. Mitochondrial DNA in inflammation and immunity. EMBO Rep. (2020) 21:e49799. doi: 10.15252/embr.201949799 32202065 PMC7132203

[30] Nguyen NNY Kim SS Jo YH. Deregulated mitochondrial DNA in diseases. DNA Cell Biol. (2020) 39:1385–400. doi: 10.1089/dna.2019.5220 31944832

[31] Salabei JK Hill BG. Mitochondrial fission induced by platelet-derived growth factor regulates vascular smooth muscle cell bioenergetics and cell proliferation. Redox Biol. (2013) 1:542–51. doi: 10.1016/j.redox.2013.10.011 PMC3836280 24273737

[32] Li J Li X Song S Sun Z Li Y Yang L. Mitochondria spatially and temporally modulate VSMC phenotypes via interacting with cytoskeleton in cardiovascular diseases. Redox Biol. (2023) 64:102778. doi: 10.1016/j.redox.2023.102778 37321061 PMC10277590

[33] Wolf. AM. MtDNA mutations and aging-not a closed case after all? Signal Transduction Targeted Ther. (2021) 6:56. doi: 10.1038/s41392-021-00479-6 PMC7873034 33563891

[34] Vadakedath S Kandi V Ca J Vijayan S Achyut KC Uppuluri S. Mitochondrial Deoxyribonucleic acid (mtDNA), maternal inheritance, and their role in the development of cancers: a scoping review. Cureus. (2023) 15:e39812. doi: 10.7759/cureus.39812 37397663 PMC10314188

[35] Antonyová V Kejík Z Brogyányi T Kaplánek R Pajková M Talianová V. Role of mtDNA disturbances in the pathogenesis of alzheimer’s and parkinson’s disease. DNA Repair. (2020) 91-92:102871. doi: 10.1016/j.dnarep.2020.102871 32502755

[36] Sazonova MA Ryzhkova AI Sinyov VV Sazonova MD Kirichenko TV Doroschuk NA. Mutations of mtDNA in some Vascular and metabolic diseases. Curr Pharm Design. (2021) 27:177–84. doi: 10.2174/1381612826999200820162154 32867647

[37] Sazonova MA Ryzhkova AI Sinyov VV Galitsyna EV Melnichenko AA Demakova NA. Mitochondrial genome mutations associated with myocardial infarction. Dis Markers. (2018) 2018:9749457. doi: 10.1155/2018/9749457 29670672 PMC5835263

[38] Vilne B Sawant A Rudaka I. Examining the association between mitochondrial genome variation and coronary artery disease. Genes. (2022) 13:1–18. doi: 10.3390/genes13030516 PMC8953999 35328073

[39] Jia Q Xu L Shen J Wei Y Xu H Shi J. Detecting rare variants and heteroplasmy of mitochondrial DNA from high-throughput sequencing in patients with coronary artery disease. Med Sci Monitor: Int Med J Exp Clin Res. (2020) 26:e925401. doi: 10.12659/MSM.925401 PMC7646198 33132382

[40] Wang Z Chen H Qin M Liu C Ma Q Chen X. Associations of mitochondrial variants with lipidomic traits in a chinese cohort with coronary artery disease. Front Genet. (2021) 12:630359. doi: 10.3389/fgene.2021.630359 33841498 PMC8027325

[41] Liu F He J Wang S Yu F Luo Z. Association of m.5178C>A variant with serum lipid levels: a systematic review and meta-analysis. Bioscience Rep. (2021) 41:1–10. doi: 10.1042/BSR20212246 PMC8685646 34859818

[42] Zhou X Wang Z Qin M Zhong S. Mitochondrial G12630A variation is associated with statin-induced myalgia in Chinese patients with coronary artery disease. Nan fang yi ke da xue xue bao = J South Med Univ. (2020) 40:1747–52. doi: 10.12122/j.issn.1673-4254.2020.12.08 PMC7835699 33380401

[43] Ashar FN Zhang Y Longchamps RJ Lane J Moes A Grove ML. Association of mitochondrial dna copy number with cardiovascular disease. JAMA Cardiol. (2017) 2:1247–55. doi: 10.1001/jamacardio.2017.3683 PMC5710361 29049454

[44] Wang XB Cui NH Zhang S Liu ZJ Ma JF Ming. L. Leukocyte telomere length, mitochondrial DNA copy number, and coronary artery disease risk and severity: A two-stage case-control study of 3064 Chinese subjects. Atherosclerosis. (2019) 284:165–72. doi: 10.1016/j.atherosclerosis.2019.03.010 30921599

[45] Luo J Noordam R Jukema JW van Dijk KW Hägg S Grassmann F. Low mitochondrial copy number drives atherogenic cardiovascular disease: evidence from prospective cohort analyses in the UK Biobank combined with Mendelian Randomization. medRxiv. (2021), 1–31. doi: 10.1101/2021.07.01.21259854

[46] Vecoli C Borghini A Pulignani S Mercuri A Turchi S Carpeggiani C. Prognostic value of mitochondrial DNA(4977) deletion and mitochondrial DNA copy number in patients with stable coronary artery disease. Atherosclerosis. (2018) 276:91–7. doi: 10.1016/j.atherosclerosis.2018.07.015 30053637

[47] Liu X Longchamps RJ Wiggins KL Raffield LM Bielak LF Zhao W. Association of mitochondrial DNA copy number with cardiometabolic diseases. Cell Genomics. (2021) 1:1–8. doi: 10.1016/j.xgen.2021.100006 PMC8758111 35036986

[48] Liu X Sun X Zhang Y Jiang W Lai M Wiggins KL. Association between whole blood-derived mitochondrial DNA copy number, low-density lipoprotein cholesterol, and cardiovascular disease Risk. J Am Heart Assoc. (2023) 12:e029090. doi: 10.1161/JAHA.122.029090 37804200 PMC10757530

[49] Yu EPK Reinhold J Yu H Starks L Uryga AK Foote K. Mitochondrial respiration is reduced in atherosclerosis, promoting necrotic core formation and reducing relative fibrous cap thickness. Arteriosclerosis Thrombosis Vasc Biol. (2017) 37:2322–32. doi: 10.1161/ATVBAHA.117.310042 PMC5701734 28970293

[50] You Y Chen X Chen Y Pang J Chen Q Liu Q. Epigenetic modulation of Drp1-mediated mitochondrial fission by inhibition of S-adenosylhomocysteine hydrolase promotes vascular senescence and atherosclerosis. Redox Biol. (2023) 65:102828. doi: 10.1016/j.redox.2023.102828 37517319 PMC10400927

[51] Chen H Vermulst M Wang YE Chomyn A Prolla TA McCaffery JM. Mitochondrial fusion is required for mtDNA stability in skeletal muscle and tolerance of mtDNA mutations. Cell. (2010) 141:280–9. doi: 10.1016/j.cell.2010.02.026 PMC2876819 20403324

[52] Ježek J Cooper KF Strich R. Reactive oxygen species and mitochondrial dynamics: the yin and yang of mitochondrial dysfunction and cancer progression. Antioxidants (Basel Switzerland). (2018) 7:1–24. doi: 10.3390/antiox7010013 PMC5789323 29337889

[53] Chakrabarti R Higgs HN. Revolutionary view of two ways to split a mitochondrion. Nature. (2021) 593:346–7. doi: 10.1038/d41586-021-01173-x PMC9813928 33953387

[54] Collins LV Hajizadeh S Holme E Jonsson IM Tarkowski A. Endogenously oxidized mitochondrial DNA induces *in vivo* and *in vitro* inflammatory responses. J Leukocyte Biol. (2004) 75:995–1000. doi: 10.1189/jlb.0703328 14982943

[55] Garcia-Martinez I Santoro N Chen Y Hoque R Ouyang X Caprio S. Hepatocyte mitochondrial DNA drives nonalcoholic steatohepatitis by activation of TLR9. J Clin Invest. (2016) 126:859–64. doi: 10.1172/JCI83885 PMC4767345 26808498

[56] Xie S Su E Song X Xue J Yu P Zhang B. GSDME in endothelial cells: inducing vascular inflammation and atherosclerosis via mitochondrial damage and sting pathway activation. Biomedicines. (2023) 11:1–15. doi: 10.3390/biomedicines11092579 PMC10526370 37761020

[57] Jin Y Liu Y Xu L Xu J Xiong Y Peng Y. Novel role for caspase 1 inhibitor VX765 in suppressing NLRP3 inflammasome assembly and atherosclerosis via promoting mitophagy and efferocytosis. Cell Death Dis. (2022) 13:512. doi: 10.1038/s41419-022-04966-8 35641492 PMC9156694

[58] Inzaugarat ME Wree A Feldstein AE. Hepatocyte mitochondrial DNA released in microparticles and toll-like receptor 9 activation: A link between lipotoxicity and inflammation during nonalcoholic steatohepatitis. Hepatol (Baltimore Md.). (2016) 64:669–71. doi: 10.1002/hep.28666 PMC4956555 27239763

[59] Li Q Wu P Du Q Hanif U Hu H Li K. cGAS-STING, an important signaling pathway in diseases and their therapy. MedComm. (2024) 5:e511. doi: 10.1002/mco2.v5.4 38525112 PMC10960729

[60] Kim J Kim HS Chung JH. Molecular mechanisms of mitochondrial DNA release and activation of the cGAS-STING pathway. Exp Mol Med. (2023) 55:510–9. doi: 10.1038/s12276-023-00965-7 PMC10037406 36964253

[61] Ueda K Sakai C Ishida T Morita K Kobayashi Y Horikoshi Y. Cigarette smoke induces mitochondrial DNA damage and activates cGAS-STING pathway: application to a biomarker for atherosclerosis. Clin Sci (London England: 1979). (2023) 137:163–80. doi: 10.1042/CS20220525 PMC9874975 36598778

[62] An C Sun F Liu C Huang S Xu T Zhang C. IQGAP1 promotes mitochondrial damage and activation of the mtDNA sensor cGAS-STING pathway to induce endothelial cell pyroptosis leading to atherosclerosis. Int Immunopharmacol. (2023) 123:110795. doi: 10.1016/j.intimp.2023.110795 37597406

[63] Grebe A Hoss F Latz E. NLRP3 inflammasome and the IL-1 pathway in atherosclerosis. Circ Res. (2018) 122:1722–40. doi: 10.1161/CIRCRESAHA.118.311362 29880500

[64] Lu N Cheng W Liu D Liu G Cui C Feng C. NLRP3-mediated inflammation in atherosclerosis and associated therapeutics. Front Cell Dev Biol. (2022) 10:823387. doi: 10.3389/fcell.2022.823387 35493086 PMC9045366

[65] Nakahira K Haspel JA Rathinam VA Lee SJ Dolinay T Lam HC. Autophagy proteins regulate innate immune responses by inhibiting the release of mitochondrial DNA mediated by the NALP3 inflammasome. Nat Immunol. (2011) 12:222–30. doi: 10.1038/ni.1980 PMC3079381 21151103

[66] Huang Y Zhou JH Zhang H Canfran-Duque A Singh AK Perry RJ. Brown adipose TRX2 deficiency activates mtDNA-NLRP3 to impair thermogenesis and protect against diet-induced insulin resistance. J Clin Invest. (2022) 132:1–45. doi: 10.1172/JCI148852 PMC9057632 35202005

[67] Shimada K Crother TR Karlin J Dagvadorj J Chiba N Chen S. Oxidized mitochondrial DNA activates the NLRP3 inflammasome during apoptosis. Immunity. (2012) 36:401–14. doi: 10.1016/j.immuni.2012.01.009 PMC3312986 22342844

[68] Zhou R Yazdi AS Menu P Tschopp J. A role for mitochondria in NLRP3 inflammasome activation. Nature. (2011) 469:221–5. doi: 10.1038/nature09663 21124315

[69] Zhong Z Umemura A Sanchez-Lopez E Liang S Shalapour S Wong J. NF-κB Restricts inflammasome activation via elimination of damaged mitochondria. Cell. (2016) 164:896–910. doi: 10.1016/j.cell.2015.12.057 26919428 PMC4769378

[70] Zhong Z Liang S Sanchez-Lopez E He F Shalapour S Lin XJ. New mitochondrial DNA synthesis enables NLRP3 inflammasome activation. Nature. (2018) 560:198–203. doi: 10.1038/s41586-018-0372-z 30046112 PMC6329306

[71] Xie D Guo H Li M Jia L Zhang H Liang D. Splenic monocytes mediate inflammatory response and exacerbate myocardial ischemia/reperfusion injury in a mitochondrial cell-free DNA-TLR9-NLRP3-dependent fashion. Basic Res Cardiol. (2023) 118:44. doi: 10.1007/s00395-023-01014-0 37814087

[72] Qiu Y Huang Y Chen M Yang Y Li X Zhang W. Mitochondrial DNA in NLRP3 inflammasome activation. Int Immunopharmacol. (2022) 108:108719. doi: 10.1016/j.intimp.2022.108719 35349960

[73] Miao R Jiang C Chang WY Zhang H An J Ho F. Gasdermin D permeabilization of mitochondrial inner and outer membranes accelerates and enhances pyroptosis. Immunity. (2023) 56:2523–2541.e8. doi: 10.1016/j.immuni.2023.10.004 37924812 PMC10872579

[74] Scherbakov D Sr. Duscha S Jr. Juskeviciene R 2nd Restelli L 3rd Frank S4 Laczko E5. Mitochondrial misreading in skeletal muscle accelerates metabolic aging and confers lipid accumulation and increased inflammation. RNA (New York N.Y.). (2020) 27:265–72. doi: 10.1261/rna.077347.120 PMC7901843 33262249

[75] Jia Z Zhang Y Li Q Ye Z Liu Y Fu C. A coronary artery disease-associated tRNAThr mutation altered mitochondrial function, apoptosis and angiogenesis. Nucleic Acids Res. (2019) 47:2056–74. doi: 10.1093/nar/gky1241 PMC6393294 30541130

[76] Ji K Wang W Lin Y Xu X Liu F Wang D. Mitochondrial encephalopathy Due to a Novel Pathogenic Mitochondrial tRNA(Gln) m.4349C>T Variant. Ann Clin Trans Neurol. (2020) 7:980–91. doi: 10.1002/acn3.51069 PMC7318088 32588991

